# Risk Assessment of Import Cold Chain Logistics Based on Entropy Weight Matter Element Extension Model: A Case Study of Shanghai, China

**DOI:** 10.3390/ijerph192416892

**Published:** 2022-12-15

**Authors:** Qiang Fu, Yurou Sun, Lei Wang

**Affiliations:** College of Transport and Communications, Shanghai Maritime University, Shanghai 201306, China

**Keywords:** Entropy Weight Matter Element Extension Model, import, cold chain logistics, safety supervision, risk assessment

## Abstract

The development of world trade and fresh-keeping technology has led to the rapid development of international cold chain logistics. However, the novel coronavirus epidemic continues to spread around the world at the present stage, which challenges disease transmission control and safety supervision of international cold chain logistics. Constructing an Import Cold Chain Logistics Safety Supervision System (ICCL-SSS) is helpful for detecting and controlling disease import risk. This paper constructs an evaluation index system of ICCL safety that comprehensively considers the potential risk factors of three ICCL processes: the logistics process in port, the customs clearance process, and the logistics process from port to door. The risk level of ICCL-SSS is evaluated by combining the Extension Decision-making Model and the Entropy Weight Method. The case study of Shanghai, China, the world’s largest city of ICCL, shows that the overall risk level of ICCL-SSS in Shanghai is at a moderate level. However, the processes of loading and unloading, inspection and quarantine, disinfection and sterilization, and cargo storage are at high risk specifically. The construction and risk assessment of ICCL-SSS can provide theoretical support and practical guidance for improving the safety supervision ability of ICCL regulation in the post-epidemic era, and helps the local government to scientifically formulate ICCL safety administration policies and accelerate the development of world cold chain trade.

## 1. Introduction

With the high-speed development of fresh-keeping technology and logistics, the quality of cold chain food and logistics service level has attracted people’s attention. Since the outbreak of COVID-19, the demand for a stable food supply and safe vaccine delivery has put forward higher requirements for the safety of international cold chain logistics (ICCL). However, disease control processes burden ICCL supervision and bring uncertainty to cold chain stability. For many countries, it is continuously a challenge to avoid COVID-19 import risks through ICCL. From the general perspective, the “cold chain break” and “fake cold chain” problems still exist, and there are many safety hazards in the ICCL process. Therefore, its safety supervision urgently needs to be strengthened. Based on the Entropy Weight Matter Element Extension Model (EWMEEM), this paper constructs a safety supervision index system by analyzing the risks in the ICCL process, and takes Shanghai as an example to evaluate the risk level of ICCL-SSS.

Cold chain logistics is an emerging logistics field that has developed rapidly in recent years. In this field, scholars have mostly focused on problems in cold storage location, the vehicle scheduling problem (VSP), and vehicle routing problem (VRP). The storage location problem focuses on building cold chain location-allocation decision models by using big data with geographical information [1,2,3]; VSP deals with solving the multi-objective vehicles scheduling model by using intelligent algorithms in the shortest time [4,5,6]; and VRP places emphasis on optimization algorithms to arrange vehicle delivery routes in cold chain logistics [7,8,9,10,11]. With governments around the world accelerating the full implementation of the carbon emissions trading mechanism, many scholars have put the minimum carbon emissions into cold chain path planning goals [12,13,14,15].

The above research mainly focuses on the system design and management of cold chain logistics. However, the process of cold chain logistics will cause safety risk problems, which need exploration in the aspect of relative supervision and evaluation. The evaluation of safety and reliability in the process of cold chain logistics transportation also plays an important role in maintaining an efficient ICCL system. Existing studies mostly focus on studying the safety of the transportation process, for example, the safety of the maritime transportation process [16] and the safety of the land transportation process [17,18]. It should be noted that the entire cold chain consists of many crucial processes, where the port cold chain process connects maritime transportation and land transportation. During the port cold chain process, the cold chain cargo needs to go through transshipment, loading and unloading, and customs clearance, which are the most complicated processes in the ICCL. Studies on the port cold chain logistics safety and risk analysis are limited at the current stage, especially under the impact of disease prevention and disease transmission control in the post-epidemic era.

In the related research of risk assessment methods, the neural network model, the fuzzy comprehensive evaluation method, and the grey correlation analysis are mostly used. Scholars have applied the neural network model to coal mine-related safety evaluation [19,20,21], network security evaluation [22,23], hoisting machinery safety evaluation [24], road safety evaluation [25], and other fields. The fuzzy comprehensive evaluation method is widely used in safety evaluation in different fields, for it can scientifically quantify the fuzzy evaluation objects and transform the fuzzy information into more accurate mathematical expressions [26,27,28,29]. Grey correlation analysis focuses on the correlation between overall safety and various factors [30,31,32,33,34]. The above studies reflect the application of risk assessment in different fields. However, the appropriate methods to quantitatively evaluate import cold chain safety have not been adequately analyzed and reported yet.

In addition, the cold chain logistics industry has not yet established a suitable cold chain logistics risk assessment system. In the related studies on logistics risk assessment, scholars focus on the safety of the domestic cold chain product operation process, and rarely mention the safety of the cold chain logistics import process, and there are few studies on quantitative evaluation of ICCL safety. Against the background of normalization of epidemic prevention and the increasing importance of cold chain security, this paper constructs ICCL-SSS indicators based on the EWMEEM, takes the case of Shanghai, China as an example, and puts forward suggestions on the construction and optimization of ICCL-SSS in Shanghai, China. The weights of the constructed evaluation index system are combined with the basic principle of information entropy, which can improve the accuracy and validity of the assigned weights as much as possible. Compared with traditional evaluation methods, the EWMEEM has certain expandability and flexibility, which can evaluate the safety supervision risk with a single index, but also evaluate multiple indexes at the same time, and quantitatively give the level status of the safety supervision risk.

The main contributions of the current work are as follows:

(1) Considering the potential risk factors of ICCL in the logistics process in port, the customs clearance process, and the logistics process from port to door, this study constructs the risk assessment index of ICCL;
(2) The Extension Decision-making Model and Entropy Weight method are combined to comprehensively evaluate the risk of ICCL;
(3) Through the analysis of the case study of Shanghai, China, it is verified that the established model is available to make suggestions for the construction and optimization of China’s Shanghai ICCL-SSS.

The results of this paper provide policy implications for the relevant government administrations to formulate ICCL safety supervision measures and promote the development of world cold chain trade under the background of the continuous spread of COVID-19, and improve the safety supervision ability of ICCL around the world.

## 2. Risk Analysis of ICCL-SSS

### 2.1. Analysis of the ICCL Process

**Figure 1:**
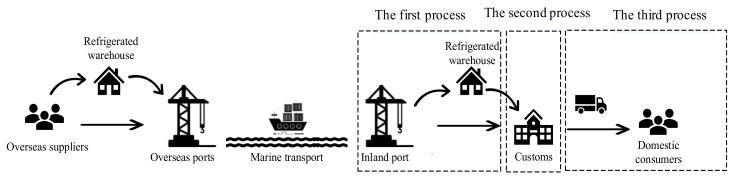
The Import Processes of International Cold Chain Logistics.

According to the current ICCL process routine, the import processes of international cold chain logistics are shown in Figure 1. In this study, considering the differences in operation roles, the import process of international cold chain logistics can be divided into three main parts: the logistics process in the port, the customs clearance process, and the logistics process from port to door. Risks in any process may eventually lead to unsafe events.

**Figure 2:**
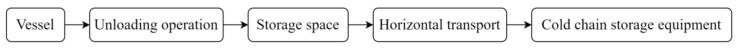
Flow chart of logistics process in port.

**Figure 3:**
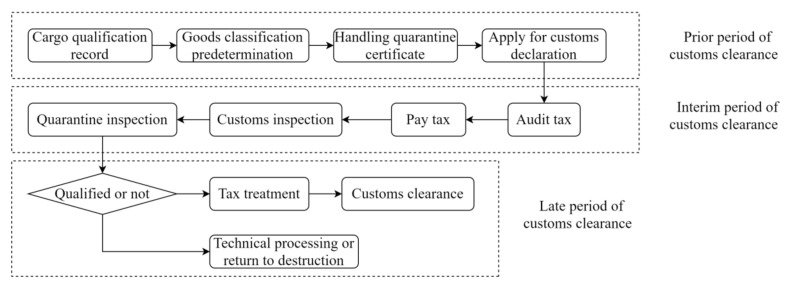
Flow chart of customs clearance process.

**Figure 4:**
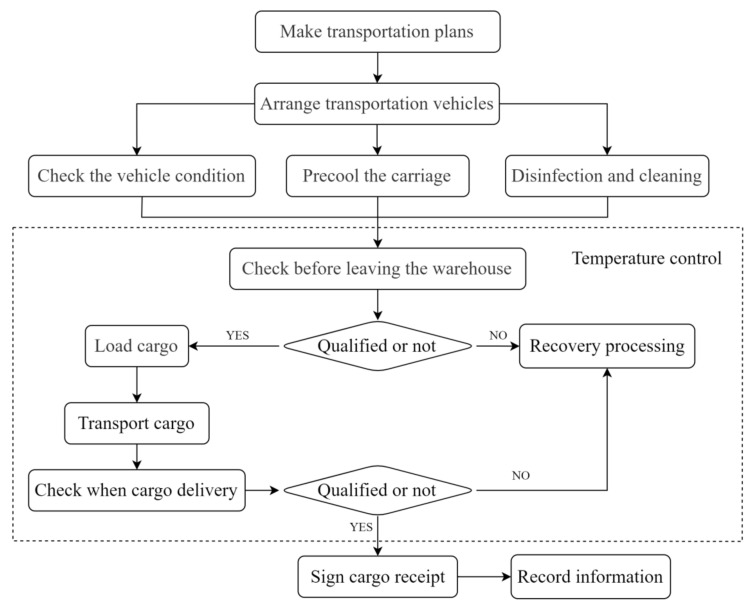
Flow chart of the logistics process from port to door.

(1) The logistics process in port. As shown in Figure 2, the cargo is first unloaded from the ship to the front yard storage space through the port unloading operation, to be transferred between the water area and the land area. Then, horizontal transportation is carried out through the vehicles in the port, and the cold chain cargo is transported to the cold chain warehouse and the inspection equipment, which will be released after customs clearance. The safety and standardization of cold chain operation in port directly determine the stability of cold chain cargo in the port’s external environment and the efficiency of operations production, thus affecting the quality of cold chain cargo and cold chain logistics costs.

(2) The customs clearance process. As shown in Figure 3, the consignee or the agent provides the carrier or Non-Vessel Operating Common Carrier (NVOCC)with the bill of lading (B/L) and corresponding information to take the cargo. Different from common cargo, cold chain cargo is classified and pre-determined with the requirement of record by customs. Then, the customs declaration application is proposed, and the customs declaration is pre-recorded. The customs declaration information is first entered into the relevant system to be submitted to the customs for a check, and a tax bill is produced according to the pre-reported content. The customs will carry out quarantine inspections on those cargos according to the corresponding testing requirements of different cargos to prevent and control foreign species and diseases carried by cargos. If the inspection is qualified, the customs shall handle the taxes paid by the consignee and release the cargo; if the customs inspection fails, the cargo will be technically processed, returned, or destroyed.

(3) The logistics process from port to door. As shown in Figure 4, after customs clearance and release, the vehicle transport plan can be formulated. The vehicle needs to carry out vehicle condition inspection, cleaning and disinfection, and pre-cooling of the carriage before transporting cold chain cargo. The carrier also needs to inspect the cargo before they are out of the warehouse. After passing the inspection, the vehicle will be loaded for transportation. After delivery and acceptance, the return receipt can be signed to complete the transportation outside the port. In this process, if the cargo is not qualified for the outbound inspection or delivery acceptance, the cargo will be recalled for processing and delivery cannot be successfully achieved. For cold chain cargos, out-of-port logistics require full temperature control to ensure the quality of cold chain goods.

### 2.2. Risk Analysis of Logistics Safety Supervision in Port

In the process of in-port logistics safety supervision, risks mainly come from the in-cabin operation, unloading process, facilities and equipment, port management level, staff quality, and port operation environment.

(1) In-cabin operational risk. Refrigerated cargos are quite sensitive to changes in temperature, humidity, and other external factors, and the quality of goods exposed to high temperature or even room temperature is highly susceptible to damage. Reefer containers need to be cut off by the ship before unloading, so the efficiency of the in-cabin operation directly affects the stability of the refrigeration environment of refrigerated cargo. There are also risks such as lifting injury and overturning of cargo when moving refrigerated cargo during in-cabin operation.
(2) Loading and unloading process risk. The risks that may occur in the loading and unloading process include (a) lifting injuries, cranes may occur in high-altitude operations, overturning, crushing, boom fall, hanging collision, braking machine failure fall and other risks; (b) fall from height, the deck or side operations, quay operations, bollard operations, machinery maintenance, on and off the ship may have fall risks; (c) machinery and equipment injuries, between the operating equipment and tools may cause hinging, rolling, touching, cutting, poking, cutting and other injuries; (d) transport injuries, vehicles in the horizontal transportation process may cause objects collapse, falling extrusion and even personnel traffic accidents; (e) drowning, in the wharf, the ship deck for cable, uncoupling and ship, and shore docking work, may occur drowning accidents.
(3) Equipment and facilities risk. (a) Cranes and other equipment in the port area of the old degree of quality will bring some security risks; (b) risk of motor vehicles in the port area, including the old degree of vehicles, the integrity of the refrigeration equipment in the vehicle, refrigeration efficiency, which will directly affect the stability of the refrigeration environment in the process of horizontal transport of cold chain cargos; (c) risk of cold chain storage equipment and facilities.
(4) Port management level risks. These risks include the degree of implementation of the responsibility for port production safety, the standardization of the implementation of port safety technical standards, the degree of supervision of port production safety, the ability to respond to emergency safety events, the degree of perfection of the emergency response system, and the degree of perfection of the emergency plan to deal with port emergencies, etc.
(5) Staff quality. The work quality of the port operation workers directly determines the orderly and efficient import cold chain in the logistics link in the port. Any misconduct of the operating staff may bring risks such as damage to equipment and even casualties.
(6) Port operation environment. This includes various hardware facilities in the port, the level of information technology, and external factors such as weather. Weather conditions, such as high-temperature environments, may cause risks such as the decay of refrigerated cargo and heat stroke of operators.

### 2.3. Risk Analysis of Customs Clearance Safety Supervision

In the process of customs clearance and safety supervision, the risks mainly come from clearance efficiency, inspection and quarantine, disinfection and sterilization, cargo storage, customs management level, and information integration risks.

(1) Customs clearance efficiency. The efficiency of customs clearance affects the length of time imported cold chain cargos remain in customs clearance, reducing the efficiency of port clearance may result in a decline in the quality of the cold chain, while low efficiency will also cause a backlog of cold chain cargos stalled in the port, which in turn leads to a lack of cold storage capacity and destroys the stability of the industrial supply chain.
(2) Inspection and quarantine risk. After accepting the customs declaration from the freight forwarder, customs will check whether the cargos and documents are consistent with the relevant provisions, including the quantity and nature, value, origin, condition, etc. of the cargo, and whether the details of the declaration have been filled in, while the actual inspection of cargos, sensory inspection, and safety sampling inspection is conducted. The risks at this point include the risk of abnormal documents and abnormal cargo.
(3) Disinfection and sterilization risk. For imported cold chain cargos, customs should conduct strict testing for COVID-19 to prevent virus importation through cold chain cargos and the risk of virus cross-infection, and the customs should organize and guide the relevant parties involved in the import of cold chain cargos, the inside of the specific product box, and the outer packaging for preventive comprehensive disinfection.
(4) Cargo storage risk. Cold chain cargos have strict requirements for storage environments, which need to be equipped with refrigeration equipment and closed facilities. In the inspection process, it is necessary to ensure that the cargos are always in a low-temperature state and maintain the relative closure of the cold chain inspection platform. At this time, once stored improperly, the quality of cold chain cargo may be damaged.
(5) Customs supervision level. The supervision level of the customs is the key to improving its customs clearance service ability, and also the key to ensuring the safety of the import cold chain. Its supervision level is mainly reflected in the supervision system, release system, and emergency linkage mechanism, as well as the management of the staff who implement the supervision.
(6) Information integration risk. The customs clearance process requires strict control of information flow to avoid information flow interruption and information traceability risk.

### 2.4. Risk Analysis of Logistics Safety Supervision from Port to Door

In the process of logistics safety supervision from port to door, the risks mainly come from transportation planning, checking before leaving the warehouse, departure preparation, cargo handling, and information recording.

(1) Transport planning. Out-port logistics need to develop a complete transportation plan, reasonably arrange transportation time, and plan transportation routes. Improper planning may face various risks such as road congestion, transportation delays, or road bumps leading to cargo damage.
(2) Checking before leaving the warehouse. After the release of cold chain cargo, it is necessary to check the quantity, type, and customer information of cold chain cargo in order to avoid errors in logistics transportation. At the same time, it is necessary to detect the temperature and humidity of cold chain cargo, and unqualified cold chain cargos need to be recycled.
(3) Departure preparation. Before transporting cold chain cargo, it is necessary to conduct a comprehensive inspection of transport vehicles, including inspection of vehicle conditions, disinfection and cleaning of compartments, and pre-cooling of compartments. In particular, it is necessary to check whether the temperature control and humidity control devices in the vehicle can work properly to ensure the safety of personnel and cargo in the cold chain transportation process.
(4) Cargo handling risk. It is necessary to improve the loading and unloading efficiency of cold chain cargos and avoid the long loading and unloading time affecting the stability of the cold chain refrigeration environment. At the same time, it is necessary to avoid risks such as the falling and crushing of cargo.
(5) Information recording risk. Complete records of relevant information should be made in all aspects of logistics outside the port, including cold chain cargo warehouse preparation records, warehouse inspection records, whole process temperature, humidity control records, whole process GPS monitoring information, and passenger and cargo acceptance records. Improper information records may lead to difficulties in accurately finding the cause of the accident when transportation problems occur, affecting information traceability and responsibility traceability.

## 3. Methodology

**Figure 5:**
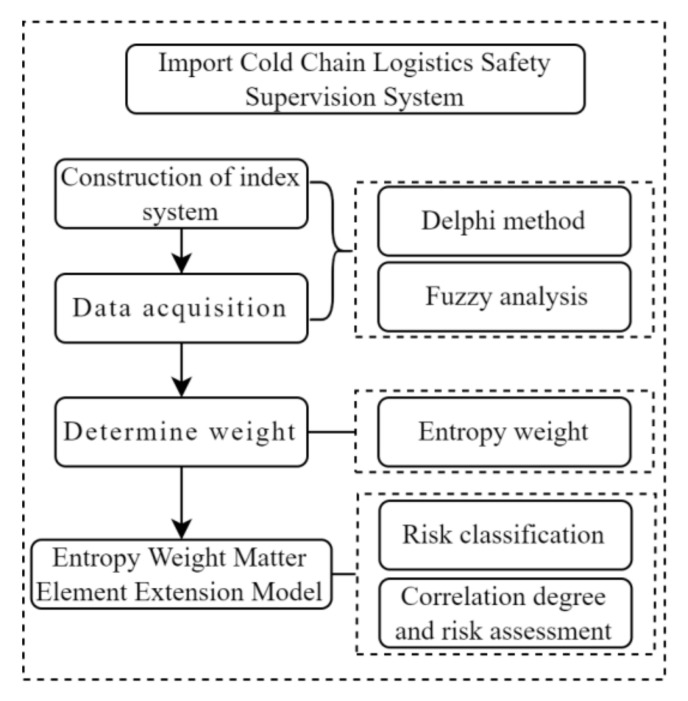
The architecture of the method.

Considering the complexity of ICCL processes, especially involving the assessment of disease transmission risk, this paper presents the EWMEEM to analyze the processing risks. Experts from the logistics field understand the operation risks from working perspectives under the impacts for disease transmission control measures, but their opinions contain subjectivity. The Delphi method is the most appropriate method to reveal the information collected from the experts. Adopting the entropy of information captures the dispersion of the opinions from different experts. The EWMEEM ensures the expandability and flexibility of the index system that newly introduced the impact from the disease transmission and other safety supervision risks. Figure 5 shows the architecture of this method.

### 3.1. Construction of Index System

Through the analysis of each process, this paper constructs the ICCL safety supervision index system as shown in Table 1. The safety supervision index of ICCL is constructed based on the whole process of ICCL. Considering the various risks that affect logistics safety in the three processes, the selected indexes are objective, feasible, and internally linked, which are in line with the integrity principle and scientific principle of the index system construction.

### 3.2. Delphi Method and Fuzzy Analysis

The Delphi method [35] is a research method that quantifies qualitative descriptions, which sets a number of indexes according to the specific requirements of the evaluation object, develops evaluation criteria based on the indexes, and invites representative experts with their own experience to evaluate the indexes. Fuzzy theory [36] is a fundamental property of events, and fuzzy analysis deals with objects through precise numerical means, which can make a more scientific, reasonable, and practical quantitative evaluation of information.

The Delphi method is used to conduct correspondence with experts to score the proposed safety supervision risk evaluation index system. The reliability of the index system is judged by the statistics of experts’ enthusiasm, the degree of experts’ authority, the degree of concentration, and coordination of experts’ opinions. The final index system was established after adjusting the indexes by combining the experts’ opinions, and the risk level of each index was expressed by precise values in combination with the fuzzy analysis. This paper pays attention to the following principles when using the Delphi method and fuzzy analysis:

(1) Invite representative and authoritative experts: inviting experts who specialize in the management and operation of cold chain logistics in logistics enterprises.
(2) Obtaining the support of participants to ensure that experts make careful evaluations of each index.
(3) Providing experts with as much information as possible and combining experts’ own experience to make judgments.

### 3.3. Entropy Weight

Entropy was originally a concept in thermodynamics, and was introduced into information theory and named information entropy [37]. It calculates the entropy weight of each index with the help of information entropy, and the weight of the index is corrected based on this value to achieve the assignment of the weight. It has been used in the quantitative analysis of problems; for example, scholars have used it to quantitatively analyze the evolution of international grain trade patterns [38]. The formula is as follows:

(1) Calculate standardized index ratios (*P_ij_*)
  (1) $$P_{ij}=\frac{X_{ij}}{\sum_{i=1}^{m}X_{ij}}$$
(2) Calculate index information entropy (*E_j_*)
  (2) $$E_{j}=-k\sum_{i=1}^{m}P_{ij}\times \ln P_{ij}$$
(3) Calculate the information entropy redundancy (*d_j_*)
  (3) $$d_{j}=1-E_{j}$$
(4) Calculation of index weights (*W_j_*)
  (4) $$W_{j}=\frac{d_{j}}{\sum_{j=1}^{n}d_{j}}$$

### 3.4. Risk Classification

This paper analyzes and delimits the risk level of ICCL safety supervision, and finally divides it into five levels. From low to high, they are the low risk (I), the medium low risk (II), the moderate risk (III), the medium high risk (IV), and the high risk (V). The single risk correlation degree and comprehensive correlation degree are calculated using each index value and index weight.

(1) Classical domain

According to the risk threshold determined by expert scoring, the classical domains *R*_01_, *R*_02_, *R*_03_, *R*_04_, and *R*_05_ of ICCL safety supervision risk are as follows:

(5) $$R_{01}=\left(\begin{array}{ccc} I & In-cabin\;operational\;risk & \left(0,2\right)\\ & \vdots & \vdots \\ & Information\;recording\;risk & \left(0,2\right) \end{array}\right)$$

(6) $$R_{02}=\left(\begin{array}{ccc} II & In-cabin\;operational\;risk & \left(2,4\right)\\ & \vdots & \vdots \\ & Information\;recording\;risk & \left(2,4\right) \end{array}\right)$$

(7) $$R_{03}=\left(\begin{array}{ccc} III & In-cabin\;operational\;risk & \left(4,6\right)\\ & \vdots & \vdots \\ & Information\;recording\;risk & \left(4,6\right) \end{array}\right)$$

(8) $$R_{04}=\left(\begin{array}{ccc} IV & In-cabin\;operational\;risk & \left(6,8\right)\\ & \vdots & \vdots \\ & Information\;recording\;risk & \left(6,8\right) \end{array}\right)$$

(9) $$R_{05}=\left(\begin{array}{ccc} V & In-cabin\;operational\;risk & \left(8,10\right)\\ & \vdots & \vdots \\ & Information\;recording\;risk & \left(8,10\right) \end{array}\right)$$

Then, the classical domain review matter element matrix of ICCL supervision risk assessment is:

(10) $$R_{0j}=\left(\begin{array}{cccccc} & I & II & III & IV & V\\ A_{1} & \left(0,2\right) & \left(2,4\right) & \left(4,6\right) & \left(6,8\right) & \left(8,10\right)\\ A_{2} & \left(0,2\right) & \left(2,4\right) & \left(4,6\right) & \left(6,8\right) & \left(8,10\right)\\ \vdots & \vdots & \vdots & \vdots & \vdots & \vdots \\ C_{5} & \left(0,2\right) & \left(2,4\right) & \left(4,6\right) & \left(6,8\right) & \left(8,10\right) \end{array}\right)$$

(2) Joint domain

According to the risk level threshold of each evaluation index, the risk joint domain of ICCL supervision is:

(11) $$R_{P}=\left(\begin{array}{cc} A_{1} & \left(0,10\right)\\ A_{2} & \left(0,10\right)\\ \vdots & \vdots \\ C_{5} & \left(0,10\right) \end{array}\right)$$

(3) Evaluation object

Based on the expert scoring data of each risk index, this paper quantitatively evaluates the status of ICCL supervision, and calculates and analyzes the risk levels of ICCL supervision, in Shanghai, China. Among them, the object to be evaluated is expressed by the Matter Element Model as follows:

(12) $$R_{0}=\left(\begin{array}{ccc} P_{0} & In-cabin\;operational\;risk & a_{1}\\ & \vdots & \vdots \\ & Information\;recording\;risk & c_{5} \end{array}\right)$$

### 3.5. Correlation Degree and Risk Assessment

This paper uses the correlation function from the extension theory. Using the following formula to calculate the correlation degree of each secondary risk index, combined with the weight of each index, the correlation degree of each primary risk index is calculated.

(13) $$K_{0j}\left(v_{i}\right)=\left\{\begin{array}{l} \frac{\rho \left(v_{i},v_{0ji}\right)}{\rho \left(v_{i},v_{pi}\right)-\rho \left(v_{i},v_{0ji}\right)},v_{i}\notin v_{0ji}\\ -\frac{\rho \left(v_{i},v_{0ji}\right)}{\left|a_{0ji}-b_{0ji}\right|},v_{i}\in v_{0ji} \end{array}\right.$$

where *K*_0*j*_(*v_i_*) denotes the correlation degree between the risk index *v_i_* and the risk level *j*; *v_ρi_* is the nodal domain; *v*_0*ij*_ is the classical domain; and *a*_0*ji*_ and *b*_0*ji*_ are the boundary values of the risk level *j*, respectively. *ρ*(*v_i_*,*v_pi_*) and *ρ*(*v_i_*,*v*_0*ji*_) denote the distance between *v_i_* and the joint domain and the classical domain, respectively.

The comprehensive correlation degree *K*_0*j*_(*R*_0_) is the object to be evaluated *R*_0_. In this case, the import safety supervision risk of Shanghai, China. For the weighted value of the correlation degree of each grade *j*, the results fully consider the influence of membership relationship and single index on the whole safety supervision evaluation system. The calculation formula is as follows:

(14) $$K_{0j}(R_{0})=\sum_{i=1}^{n}w_{i}K_{0j}(v_{i})$$

where *w_i_* and *v_i_* are the weight and index value of each evaluation index, respectively.

## 4. Case Study

### 4.1. Data Source

In this paper, 12 experts from a logistics enterprise in Shanghai, China were invited to evaluate the risk of ICCL safety supervision in Shanghai. The logistics enterprise has rich experience in cold chain logistics service and is one of the largest professional logistics enterprises in China, and it accounts for a large proportion (more than 80%) of the cold chain activities imported by Shanghai. Its service network covers China and even overseas. The selected experts are composed of risk assessment department personnel, cold chain logistics department management personnel, and cold chain logistics department operators. Expert scoring rules for each index are as follows: (1) the highest score of each index is 10 points, and the higher the score is, the higher the risk is considered; (2) full score of 10 points, divided into five risk levels, 0–2 for the low risk, 2–4 for the medium-low risk, 4–6 for the general risk, 6–8 for the medium-high risk, 8–10 for the high risk; (3) the average value of each index is used as the index score after scoring based on data and practical experience. In the process of data processing, combined with the Delphi method and fuzzy analysis, the final index score results are shown in Table 2 below.

To verify the reliability of the data, this paper calculated the sample coefficient of dispersion [39]. Equation (15) shows the calculation formula, and the results are shown in Table 3. It can be seen that the coefficient of dispersion of most indices is between 0.5 and 0.8, which means the indices are somewhat discrete and not completely homogeneous, indicating that all indicators are relatively well represented.

(15) $$Dr=\frac{k}{k-1}(1-\sum_{j=1}^{k}f_{rj}^{2})$$

where 0 ≤ *D_r_*≤ 1, *D_r_* is the coefficient of dispersion, *k* is the number of categories distinguished in *r* question, and *f_rj_* is the incidence of *j* category in *r* question.

### 4.2. Weight Calculation

According to the experts’ assessment reports of ICCL safety supervision in Shanghai obtained in Section 3.1, the evaluation index value matrix is standardized. In this paper, the information entropy method is used to determine the weight of each index, and the weight of the evaluation index of ICCL safety supervision in Shanghai is shown in Table 4.

### 4.3. Results and Analysis

#### 4.3.1. Calculation of Correlation Degree of First Grade Indexes

Through the calculation of this formula, the correlation degree of each level of risk index is shown in Table 5. It can be seen from the table that max *K*_0*j*_(*A*) = 0.08, max *K*_0*j*_(*B*) = 0.06, and *K*_0*j*_(*C*) = 0.07, which are all in the risk level III, that is, the port logistics safety supervision, customs clearance safety supervision, and port logistics safety supervision are all in the general risk level.

#### 4.3.2. Correlation Analysis of Secondary Indexes

To comprehensively analyze the risk degree of each secondary index, the correlation degree of each risk index was analyzed, and Table 6 is the risk level table of ICCL safety supervision risk assessment index.

According to Shanghai ICCL safety supervision single risk index analysis of existing safety supervision risk. It can be seen from Table 6 that the port management level of indicator A_4_ is in the risk level II, namely, at a medium-low risk. It indicates that the safety supervision of Shanghai Port is effective at the port management level, a relatively complete safety technical standard and emergency response system have been formulated, and more attention has been paid to the guidance and supervision of port safety production.

Among them, indicators A_1_, A_3_, A_5_, A_6_, B_1_, B_5_, B_6_, C_1_, C_2_, C_3_, C_4_, and C_5_, namely the in−cabin operational risk, equipment and facilities risk, staff quality, port operation environment, customs clearance efficiency, customs supervision level, information integration risk, transportation planning risk, risk of checking before leaving the warehouse, risk of departure preparation, cargo handling risk, information recording risk indicators are in the risk level III, namely the general risk state. This level shows that the safety supervision of the logistics process inside and outside the port needs to be improved. Cold chain logistics requires high-quality equipment and facilities, professional skills of staff, information technology, and so on. At present, the industry has not yet built an efficient and complete supervision mechanism that can restrict the whole industry.

Indicators A_2_, B_2_, B_3_, and B_4_, namely unloading process risk, inspection and quarantine risk, disinfection and sterilization risk, and cargo storage risk indicators are in the risk level IV, namely the medium-high risk. The reasons can be attributed to the following aspects. Firstly, the operation of the port handling process is complex, and there are many types of accidents involved. In addition, the port handling link lacks a unified regulatory standard, and the cold chain cargos, as special cargos, have more stringent normative requirements for the handling process. Therefore, this indicator causes a high risk of imported cold chain logistics security. Against the background of normalization of epidemic prevention and control, the risk of virus input with imported cold chain cargo is still high. The customs clearance process urgently needs to strengthen the supervision of inspection and quarantine, disinfection, and sterilization processes. At present, a complete supervision system has not been established. In the process of cold chain product clearance, the failure of refrigeration equipment and cover sealing facilities or improper storage leads to the loss of refrigerated cargo. It is necessary to further strengthen the supervision mechanism.

#### 4.3.3. Comprehensive Correlation and Risk Level

The comprehensive correlation degree under each risk level is finally calculated by Formula 14. The results are shown in Table 7.

In all *K*_0*j*_(*R*_0_) values to find out the maximum value is $\max\underset{j=1,2,\ldots 5}{K_{0j}(R_{0})}=0.21$, which can judge that the Shanghai ICCL safety supervision risk corresponding *j* = 3, which is in the risk level III, namely moderate risk.

## 5. Conclusions and Discussion

Based on the whole process of ICCL, this paper constructs the risk assessment system of ICCL safety supervision according to the main risks of each link of safety supervision, uses the EWMEEM to quantitatively study the risk problem, and takes Shanghai, China as an example to carry out the case analysis. The results are as follows:

(1) According to the comprehensive evaluation results, the safety supervision of ICCL in Shanghai is at moderate risk, and the results are in line with the current safety supervision status of ICCL in Shanghai.
(2) According to the assessment of single risk factors in a different process, the four indexes of unloading process risk, inspection and quarantine risk, disinfection and sterilization risk, and cargo storage risk are at medium-high risk. Shanghai should pay attention to the following issues in the supervision of ICCL safety.
  (a) Timely updating of cold chain handling, storage, transportation, and other technical equipment. The improper storage of cargo in customs clearance leads to a high risk of cargo damage, and the storage and transportation of imported cold chains in port logistics also have certain risks. Considering that the storage of imported cold chain cargo needs to strictly control the ambient temperature and humidity, the relevant departments should strengthen the inspection and control of cold chain technology and equipment, and pay attention to the renewal and introduction of related facilities and equipment technology.
  (b) Control of import cold chain inspection and quarantine risks and disinfection and sterilization risks. Actively cooperate with relevant departments to carry out the collection of imported cold chain food samples and the collection of nucleic acid samples from transport vehicles and employees. When the risk occurs, actively cooperate with relevant departments to conduct import cold chain food traceability management and emergency disposal work.
  (c) Establishing a unified and standardized information-sharing platform for the ICCL supply chain, and promoting the establishment of an integrated management system for the ICCL supply chain. To construct a traceability system for the whole process of the supply chain with traceable sources, traceable destinations, and accountability, improve the information sharing mechanism. In addition, provide a comprehensive and non-dead angle tracking and supervision system for the whole process of the supply chain, such as cargo storage and transportation.
  (d) Explore various forms of cold chain logistics node combinations, such as single-point, circular, and cross-type, to form an efficient transportation network and carry out the extended service of imported cold chain logistics. Vigorously develop new technologies and equipment such as automatic sorting and intelligent storage systems from aspects of management organization, distribution mode, and technical means. Explore the use of advanced equipment and technology such as distribution robots, in the “most dangerous place” instead of traditional manual distribution, through the development of intelligent, non-contact, and other new technologies to drive cold chain logistics transformation and upgrading.

Facing the post-epidemic era, this paper comprehensively sorts and summarizes the relevant experience of the prevention and control of the novel coronavirus epidemic; deeply analyzes the weak parts of the ICCL; systematically carries out the construction and optimization of the supervision system of the ICCL; discusses the construction of the supervision system of the intra-port operations, customs clearance operations, and out-port operations in the process of the ICCL supply chain; and puts forward the establishment of a unified and standardized information-sharing platform for the ICCL supply chain, so as to improve the safety supervision ability of the ICCL throughout the international cold chain logistics transportation process in the post-epidemic era. It can provide theoretical support and practical guidance for the relevant departments of local governments to formulate scientific safety supervision policies of ICCL and promote the development of the world cold chain trade.

## Figures and Tables

**Table 1:** ICCL safety supervision index system.

First Index	Second index	Remark
In-port logistics safety supervision A	In−cabin operational risk A_1_	In-cabin refrigeration A_11_, Cargo movement in the cabin A_12_
	Unloading process risk A_2_	Lift injury A_21_, High fall A_22_, Equipment injury A_23_, Transport injury A_24_, Drowning A_25_
	Equipment and facilities risk A_3_	Crane Machinery Quality A_31_, Port Vehicle Aging A_32_, Cold Chain Storage Equipment Risk A_33_
	Port management level A_4_	Safety Technology Standard A_41_, Emergency Response System A_42_, Safety Production Guidance and Supervision A_43_
	Staff quality A_5_	Staff operation strict specification A_51_
	Port operating environment A_6_	Perfect hardware facilities A_61_, Informatization level A_62_, Weather conditions on working day A_63_
Customs clearance safety supervision B	Customs clearance efficiency B_1_	Cold chain product storage shortage backlog stagnation port B_11_
	Inspection and quarantine B_2_	Document exception risk B_21_, Abnormal risk of cargos B_22_
	Disinfection and sterilization B_3_	Disinfection of inner wall and outer packing of cargos B_31_, Input of new coronavirus pneumonia B_32_, Virus cross infection B_33_
	Cargo storage risk B_4_	Facility failure of refrigeration equipment B_41_, Improper storage leads to the loss of refrigerated cargos B_42_
	Customs supervision level B_5_	Regulatory regime B_51_, Release system B_52_, Emergency response mechanism B_53_, Staff oversight B_54_
	Information integration risk B_6_	Strict control of information flow B_61_
Logistics safety supervision from port to door C	Transportation planning risk C_1_	Traffic congestion delays C_11_, Road bump risk C_12_
	Check before leaving the warehouse C_2_	Information checking of cold chain cargos C_21_, Temperature and humidity inspection of cargos C_22_
	Risk of departure preparation C_3_	Inspection of interior temperature and humidity control equipment C_31_, Disinfection and cleaning of carriage C_32_, Precooling of carriage C_33_
	Cargo handling risk C_4_	Efficiency of cargo-handlinC_41_, Falling pressure loss of cargos during loading and unloading C_42_
	Information recording risk C_5_	Information record of out-port logistics C_51_

**Table 2:** Scores of ICCL safety supervision index.

Risk index	A_1_	A_2_	A_3_	A_4_	A_5_	A_6_
Value	5.33	6.56	5.33	3.78	4.44	5.00
Risk index	B_1_	B_2_	B_3_	B_4_	B_5_	B_6_
Value	5.22	6.22	6.89	6.11	4.67	5.00
Risk index	C_1_	C_2_	C_3_	C_4_	C_5_	
Value	5.11	5.89	4.56	5.44	4.89	

**Table 3:** Coefficient of dispersion of ICCL safety supervision index.

Risk index	A_1_	A_2_	A_3_	A_4_	A_5_	A_6_
Coefficient of dispersion	0.74	0.77	0.69	0.61	0.54	0.74
Risk index	B_1_	B_2_	B_3_	B_4_	B_5_	B_6_
Coefficient of dispersion	0.55	0.47	0.44	0.60	0.61	0.64
Risk index	C_1_	C_2_	C_3_	C_4_	C_5_	
Coefficient of dispersion	0.57	0.52	0.56	0.70	0.36	

**Table 4:** Weights of Shanghai ICCL Safety Supervision index.

Risk index	A_1_	A_2_	A_3_	A_4_	A_5_	A_6_
Weight	0.071	0.047	0.058	0.063	0.090	0.055
Risk index	B_1_	B_2_	B_3_	B_4_	B_5_	B_6_
Weight	0.086	0.066	0.051	0.062	0.063	0.055
Risk index	C_1_	C_2_	C_3_	C_4_	C_5_	
Weight	0.036	0.038	0.062	0.057	0.040	

**Table 5:** Risk Level of Shanghai ICCL Safety Supervision factors.

Factors	*j* = 1	*j* = 2	*j* = 3	*j* = 4	*j* = 5	Risk Level	Degree of Risk
*K*_0*j*_(*A*)	−0.15	−0.06	0.08	−0.06	−0.15	Ⅲ	General risk
*K*_0*j*_(*B*)	−0.18	−0.11	0.06	0.00	−0.13	Ⅲ	General risk
*K*_0*j*_(*C*)	−0.09	−0.05	0.07	−0.04	−0.09	Ⅲ	General risk

**Table 6:** Risk level of ICCL safety supervision index.

Risk index	A_1_	A_2_	A_3_	A_4_	A_5_	A_6_
Maximum	0.33	0.28	0.33	0.11	0.22	0.5
Risk level	Ⅲ	Ⅳ	Ⅲ	Ⅱ	Ⅲ	Ⅲ
Risk index	B_1_	B_2_	B_3_	B_4_	B_5_	B_6_
Maximum	0.39	0.11	0.44	0.03	0.33	0.5
Risk level	Ⅲ	Ⅳ	Ⅳ	Ⅳ	Ⅲ	Ⅲ
Risk index	C_1_	C_2_	C_3_	C_4_	C_5_	
Maximum	0.44	0.06	0.28	0.28	0.44	
Risk level	Ⅲ	Ⅲ	Ⅲ	Ⅲ	Ⅲ	

**Table 7:** Comprehensive relevance degree under each risk level.

Risk Index	*j* = 1	*j* = 2	*j* = 3	*j* = 4	*j* = 5
*K*_0*j*_(*R*_0_)	−0.42	−0.21	0.21	−0.10	−0.38

## Data Availability

This study did not report any data.
